# Beyond the Usual Suspects: Emerging Associations Between Epstein‐Barr Virus Infection/Infectious Mononucleosis and Cancers

**DOI:** 10.1002/rmv.70153

**Published:** 2026-04-25

**Authors:** Marisa D. Muckian, Graham S. Taylor, John Diaz‐Decaro, Enejda Senko, Helen R. Stagg

**Affiliations:** ^1^ London School of Hygiene & Tropical Medicine London UK; ^2^ University of Birmingham Birmingham UK; ^3^ ModernaTX Inc. Cambridge Massachusetts USA

**Keywords:** cancer, epstein‐barr virus, glandular fever, infectious mononucleosis

## Abstract

Epstein‐Barr virus (EBV) is a ubiquitous herpesvirus and a causal factor for Burkitt Lymphoma (BL), Hodgkin Lymphoma (HL), gastric carcinoma (GC) and nasopharyngeal carcinoma (NPC). Whether EBV contributes to a wider spectrum of cancers remains uncertain. We reviewed MEDLINE, Embase and Web of Science on 30^th^ July 2024 to identify observational studies that examined the association between EBV infection or EBV‐infectious mononucleosis (IM) and cancers beyond BL, HL, GC and NPC. Evidence was appraised using the Grading of Recommendations Assessment, Development and Evaluation (GRADE) framework. For cancers meeting minimum thresholds we assessed etiologic fractions (EFs), biological plausibility, epidemiological burden, latency after IM, and predictive biomarkers. Thirty‐three eligible studies were identified, yielding 13 hypotheses (i.e., specific potential reported associations between EBV infection or IM and individual cancer types) that advanced through GRADE. Breast cancer and NHL had the greatest weight of biological plausibility, cervical and prostate the least. Despite an array of tests, testicular cancer studies provided limited evidence. EFs ranged between 12.3% (IM‐breast) and 85.1% (EBV infection‐NHL). Breast and prostate cancers had the highest global incidence. Only one study (for NHL) provided data on time from IM to cancer onset, and prostate‐specific antigen was the only biomarker identified. In this review, we highlight eight cancers across six cancer groups (breast, cervical, leukaemia/other haematologic, NHL, prostate, testicular) with some evidence of EBV involvement. These results reinforce the potential long‐term value of EBV vaccine development, while emphasising the need for high‐quality prospective studies with robust methods of viral detection to establish causality.

**Abbreviations**
ALL: acute lymphoblastic leukaemia
AML: acute myeloid leukaemia
ASIR: age standardised incidence rate
AUC: area under the curve
BALF5: BamHI‐A fragment containing the fifth leftward open reading frame
BamHI: a restriction enzyme derived from Bacillus amyloliquefaciens H
BHLF: BamHI H fragment leftward open reading frame
BHRF: BamHI H fragment rightward open reading Frame
BL: Burkitt Lymphoma
BMR: Basal Metabolic Rate
CI: confidence interval
CLL: chronic lymphocytic leukaemia
CML: chronic myeloid leukaemia
CPRD: Clinical Practice Research Datalink
DCIS: ductal carcinoma in situ
DLBCL: diffuse large B‐cell lymphoma
EA: early antigen
EBER: Epstein‐Barr Virus‐Encoded Small RNAs
EBNA: Epstein‐Barr Virus Nuclear Antigen
EBV: Epstein‐Barr Virus
EBV‐W: Epstein‐Barr Virus W region DNA
EF: etiologic fraction
FL: Follicular Lymphoma
GBD: Global Burden of Disease
GC: gastric carcinoma
GLOBOCAN: Global Cancer Observatory
Gp: glycoprotein
GRADE: Grading of Recommendations Assessment, Development and Evaluation
HL: Hodgkin Lymphoma
HR: hazard ratio
IgG: immunoglobulin G
IHC: immunohistochemistry
IHME: Institute for Health Metrics and Evaluation
IL: interleukin
IM: infectious mononucleosis
ISH: in situ hybridisation
LMP: latent membrane protein
ML: malignant lymphoma
MM: multiple myeloma
MS: Multiple Sclerosis
mT/NK: Mature peripheral T‐cell and NK‐cell Lymphomas
N/A: Not Applicable
NASBA: nucleic acid sequence‐based amplification
NHL: non‐Hodgkin Lymphoma
NK: natural killer
NPC: nasopharyngeal carcinoma
OR: odds ratio
PAF: population attributable fraction
PCR: polymerase chain reaction
PEO: Population, Exposure, Outcome
PLL: prolymphocytic leukaemia
PRISMA: Preferred Reporting Items for Systematic Reviews and Meta‐Analyses
PSA: prostate‐specific antigen
RR: risk ratio
SIR: standardised incidence ratio
SLL: small lymphocytic lymphoma
UK: United Kingdom
USA/US: United States of America
VCA: viral capsid antigen
vIL: viral interleukin
ZEBRA: BamH1 Z Epstein‐Barr Virus Replication Activator

## 1 Introduction

Epstein‐Barr virus (EBV) is a herpesvirus that infects 95% of the global population for life, with different ages at infection in different populations [1]. EBV has long been known to be associated with four malignancies‐ Burkitt Lymphoma (BL), Hodgkin Lymphoma (HL), gastric carcinoma (GC) and nasopharyngeal carcinoma (NPC) [2]. These four cancers are also associated with different stages of the EBV lifecycle: type I latency is seen in BL, type I/II in GC, and type II in HL and NPC [3]. In some instances, the association between EBV and the cancer is thought to at least partly be through EBV‐infectious mononucleosis (EBV‐IM), which typically occurs in adolescence and young adulthood [4].

Recent innovations in vaccine technologies and a contemporary study strongly implicating EBV in the development of multiple sclerosis (MS) [5] has reignited the search for an EBV vaccine. Equally, the potential for a vaccine that either prevents EBV infection or EBV‐associated sequelae has stimulated interest in which other conditions such a vaccine could prevent.

In this review, we systematically examined evidence for associations between EBV infection or EBV‐IM and malignancies beyond BL, HL, GC, and NPC. We appraised the strength of evidence using the GRADE framework, estimated etiologic and population attributable fractions (EFs/PAFs) where possible, and explored biological plausibility through molecular detection studies. We also characterised the global and country‐specific burden of these cancers, examined the timing between EBV‐IM and cancer onset, and reviewed available biomarkers that might inform early detection or risk stratification.

## 2 Methods

To explore the potential role of EBV infection or EBV‐IM in cancers beyond BL, HL, GC, and NPC, we adopted a staged approach. We began with a literature review, accompanied by a quality assessment, to identify studies reporting associations between EBV infection or EBV‐IM and malignancies outside the four established EBV‐related cancers. From these studies, we extracted hypotheses, that is specific potential reported associations between EBV infection or EBV‐IM and individual cancer types and appraised their strength using the Grading of Recommendations Assessment, Development and Evaluation (GRADE) framework [6]. Only hypotheses that achieved at least a low level of certainty (the highest rating possible for observational evidence) were advanced to subsequent stages.

For the hypotheses that were advanced, we conducted targeted searches of the molecular evidence to assess biological plausibility. We also examined the global and country‐specific burden of disease using GLOBOCAN [7] and the Global Burden of Disease (GBD) Study (IHME) [8] data. From the original literature review, we extracted information on latency periods between EBV‐IM and cancer onset, where available. We also calculated PAFs and EFs using effect estimates reported in eligible studies. Finally, we performed targeted searches for biomarkers that might predict the development of EBV‐associated malignancies before clinical onset. The detailed methods for each step are described in the following sections.

### 2.1 Literature Review

#### 2.1.1 Search Strategy and Inclusion and Exclusion Criteria

We searched MEDLINE, Embase, and Web of Science on 30^th^ July 2024, using terms related to EBV infection, infectious mononucleosis (IM), and malignancy (Appendix 1). Eligible studies were defined using a population–exposure–outcome (PEO) framework (Table 1), with detailed inclusion and exclusion criteria provided in Table 2. We did not require IM to be explicitly confirmed as EBV‐related, given that EBV accounts for the majority of IM [9]. In this paper, we thus refer to IM rather than EBV‐IM where the former term is more appropriate.

**TABLE 1:** PEO framework definitions.

PEO framework item	Definition for the review
Population	Human populations globally, with no restrictions on age, sex, or geography.
Exposure	Evidence of EBV infection (serology or DNA detection) or a recorded history of IM.
Outcome	Any cancer diagnosis other than the four well‐established EBV‐associated cancers (BL, HL, NPC, GC).

*Note:* Population, exposure and outcome (PEO) framework used for the review.

Abbreviations: BL, Burkitt lymphoma; EBV, Epstein‐Barr virus; GC, gastric carcinoma; HL, Hodgkin lymphoma; IM, infectious mononucleosis; NPC, nasopharyngeal carcinoma.

**TABLE 2:** Inclusion and exclusion criteria for screening.

	Inclusion	Exclusion
Language	Studies in any language	
Time	Studies from any time point	
Study type	Original research articles Cross sectional Cohort Case‐control	Case reports Retracted studies Molecular biology studies Review articles, including systematic reviews and meta‐analyses
Study population	Human studies	Animal studies Population was those with cancer only (no healthy controls) Only documented cancer outcomes HL, BL, NPC, or GC
EBV infection or IM	EBV serostatus determined via serology, or EBV DNA detected in blood IM self‐reported or determined via medical records	EBV infection papers without a seronegative baseline (i.e., where only levels of antibodies amongst those seropositive were compared)
Ascertainment of cancer status	Self‐report Medical records or cancer registries	

*Note:* Papers retrieved from the search were screened against the following inclusion and exclusion criteria. When serological data were used to identify cases of EBV infection, we did not extract any accompanying results from nucleic acid based viral detection assays such as PCR.

Abbreviations: BL, Burkitt lymphoma; EBV, Epstein‐Barr virus; GC, gastric carcinoma; HL, Hodgkin lymphoma; IM, infectious mononucleosis; NPC, nasopharyngeal carcinoma; PCR, polymerase chain reaction.

#### 2.1.2 Screening and Data Extraction

After deduplication in EndNote, records were managed using Rayyan [10]. Title/abstract and full text screening were performed by one reviewer (MDM), with a plan to assess foreign language articles in collaboration with fluent speakers. For studies reporting on multiple cancer outcomes, all relevant data were extracted.

In addition to the criteria listed in Table 2, we also excluded papers/data for lymphomas where lymphoma subtype was not reported due to concerns about inclusion of HL. However, studies that included B‐cell lymphomas were retained, even if some cases involved BL, as BL itself is rare in most countries and these studies often reported on other non‐Hodgkin lymphomas (NHLs) that were of interest to our analysis. Data extraction was performed in duplicate (MDM, HRS) using a predefined template, with disagreements resolved by consensus. There were no foreign language papers eligible for data extraction.

#### 2.1.3 Quality Assessment

Study quality was assessed using an adapted version of the Newcastle‐Ottawa tool [11], which assesses the quality of non‐randomised studies (Table 3‐ cohort studies and Table 4‐ case control studies). The guidance of Deeks et al. [12] was used for the adaptation, which included additional questions around the appropriateness of the statistical methods used. Application of the tool was done in duplicate by MDM and HRS and disagreements were resolved by consensus.

**TABLE 3:** Modified Newcastle‐Ottawa quality assessment criteria for cohort studies.

Newcastle‐Ottawa criteria (maximum score)	Interpretation (basis for score)
Representativeness of the exposed cohort (1)	Exposed cohort representative of the underlying population targeted by the study
Selection of the non‐exposed cohort (1)	Non‐exposed cohort taken from the same underlying population as the exposed cohort
Ascertainment of the exposure (1)	Exposure measured using serology or taken from medical records
Outcome absent at start of study (1)	Cancer only diagnosed after EBV infection was ascertained or IM occurred.
Comparability of cohorts (2)	Exposed and unexposed cohorts were balanced by age and sex +1 Exposed and unexposed cohorts were also balanced by additional factors +2
Assessment of outcome (1)	Diagnosis of cancer was taken from record linkage or assessed without knowledge of the exposure
Duration of follow‐up (1)	Follow‐up was a minimum of 10 years
Adequacy of follow‐up (1)	Loss to follow up was 25% or less and balanced for both the exposed and unexposed groups. If specific numbers were not available, judgement calls were made as to whether the degree of losses appeared to be below the threshold
Appropriate statistical methods (1)	Statistical method applied was appropriate for the hypothesis and study design
Control of confounding (1)	Analysis accounted for confounding (age, sex)

*Note:* Criteria outlined by the Newcastle‐Ottawa quality assessment tool. Modifications were made according to the recommendations by Deeks et al. [12] and thus the criteria include two additional questions assessing the appropriateness of the statistical methods and controlling for confounding.

Abbreviations: EBV, Epstein‐Barr virus; IM, infectious mononucleosis.

**TABLE 4:** Modified Newcastle‐Ottawa quality assessment criteria for case‐control studies.

Newcastle‐Ottawa criteria (maximum score)	Interpretation (basis for score)
Adequacy of case definition (1)	Independent validation of cases (e.g., hospital records, registry data)
Representativeness of the cases (1)	No risk of selection bias e.g., all the cases at the hospital included
Selection of controls (1)	Community controls
Definition of controls (1)	Confirmation no cancer at baseline
Comparability of cases and controls (2)	Cases and controls were balanced by age and sex +1 Cases and controls were also balanced by additional factors +2
Ascertainment of exposure (1)	Exposure measured using serology or taken from medical records
Same method for cases and controls (1)	Same method of exposure ascertainment was used for cases and controls
Non‐response rate (1)	Similar proportion of cases and controls did not agree to be part of the study
Appropriate statistical methods (1)	Statistical method applied appropriate for the hypothesis and study design
Control of confounding (1)	Analysis accounted for confounding (age, sex)
Simultaneous ascertainment of exposure and outcome (−1)	If the exposure was measured at the same time as the outcome or, even, after it (e.g., during cancer treatment)

*Note:* Criteria outlined by the Newcastle‐Ottawa quality assessment tool. Modifications were made according to the recommendations by Deeks et al. [12] and thus the criteria include two additional questions assessing the appropriateness of the statistical methods and controlling for confounding. An additional modification was made assessing whether exposure and outcome status were determined simultaneously.

#### 2.1.4 Registration

This review was registered with PROSPERO‐ CRD42025647912. MDM is the guarantor of the work.

### 2.2 Strength of Evidence Appraisal (GRADE)

For each EBV‐cancer hypothesis identified, we applied the GRADE framework to assess certainty of evidence. In accordance with GRADE guidance, evidence from observational studies begins at low certainty and can be downgraded or upgraded depending on study characteristics. Downgrading was applied for risk of bias, inconsistency, indirectness, or imprecision; upgrading was possible for large effect sizes, dose‐response relationships, or the plausible residual confounding in an analysis only possibly leading to an underestimate of the apparent treatment effect. For our analysis, after applying the framework, hypotheses rated at least low certainty were advanced. To maintain comparability, EBV and IM hypotheses were advanced in parallel when at least one met the threshold criteria (i.e., low certainty). Breast cancer was also retained post hoc, based on prior systematic review evidence supporting its plausibility [13].

### 2.3 Biological Plausibility

For advanced hypotheses, we conducted separate, targeted, PubMed searches for molecular studies assessing EBV presence in human tumour tissue. Following the criteria from a prior review [13], we classified evidence combining three considerations together‐the types of test used, whether samples tested positive, and the number of papers. Polymerase chain reaction (PCR) evidence alone was considered weakest (although the test has the benefit of being highly sensitive) because it cannot exclude bystander EBV‐infected cells infiltrating the tumour. By comparison, in situ hybridisation (ISH) can localise EBV to tumour cells and, when performed using probes specific for the highly abundant EBV‐encoded Epstein‐Barr Virus‐Encoded Small RNAs (EBERs), can detect EBV cells regardless of their stage of viral latency or whether they are undergoing lytic replication. While immunohistochemistry (IHC) can also localise EBV to tumour cells, it is less sensitive than EBER‐ISH and antibodies specific for some EBV proteins may not detect all forms of viral latency. Combinations of PCR plus ISH plus/minus immunohistochemistry (IHC) within a single study were considered the best evidence. The presence of multiple papers provided additional reassurance‐provided that test results were positive‐particularly if only a single study had used PCR plus ISH plus/minus IHC, but other studies also provided ISH plus/minus IHC evidence.

### 2.4 PAFs and EFs

For advanced hypotheses and where adjusted effect estimates were available, we calculated PAFs (percentage of cases in the population attributable to EBV infection or IM) using Miettinen/Flegal's formula [14, 15, 16] and EFs (percentage of cases among the exposed attributable to EBV infection or IM) using standard approaches for case‐control studies [17]. Calculations were limited to studies that adjusted for at least age and sex. Adjusted odds ratios (ORs), risk ratios (RRs), or hazard ratios (HRs) from eligible studies served as inputs for EF and PAF calculations. Studies of EBV infection were required to report using either IgG‐viral capsid antigen (VCA) or IgG‐EBV nuclear antigen (EBNA) [18], as these are indicative of recent or past infection. If a study presented results for multiple relevant antibody/antigen combinations, all were extracted. If a mixture of antigen/antibody combinations were reported, these were extracted so long as at least one relevant combination was included.

### 2.5 GLOBOCAN and IHME

We obtained global and country‐specific incidence data for the advanced hypotheses from GLOBOCAN (2022) and the GBD Study (2021) using the most recent releases available from each source. Both datasets provide age‐standardised incidence rates by cancer type and country, but rely on different modelling approaches; therefore, data from both were extracted to provide complementary estimates of global burden.

### 2.6 Length of Time Until Onset of Cancer

For hypotheses involving IM, we extracted data on the time interval between IM diagnosis and subsequent cancer onset from eligible studies identified in the initial literature review. Timing data were only available and analysed for advanced IM‐related hypotheses.

### 2.7 Biomarkers

To identify biomarkers predictive of cancer development before clinical onset (i.e., that are useful for the early detection of those cancers), separate targeted PubMed searches were conducted. Inclusion criteria comprised studies reporting on large population‐based cohorts, and‐when available‐quantifiable diagnostic performance metrics, such as sensitivity, specificity, or the area under the curve (AUC) were sought. This assessment was performed only for advanced hypotheses.

## 3 Results

### 3.1 Literature Review Search Results

**FIGURE 1:**
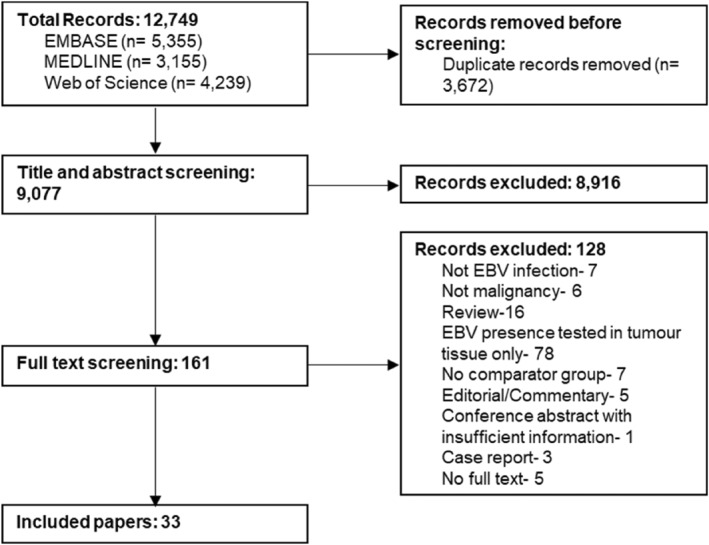
PRISMA diagram of included studies. PRISMA diagram charts the number of records excluded at the different stages of the screening process.

After searching three databases and deduplication, the literature search returned 9077 papers, of which 33 met our inclusion criteria (Figure 1, Appendix 2).

Studies were present from all world regions. Twenty‐seven (27/33, 82%) were case‐control designs and six (18%) were cohort studies. Twenty‐four (73%) examined EBV infection and nine (27%) examined IM. One (3%) reported infection results for EBV DNA only. Several studies specifically examined seroconversion following EBV infection (e.g., Cox et al. [19]), while others used IgM to detect recent EBV infection (e.g., Dagash et al. [20]). The cancers investigated ranged from breast cancer to leukaemia; some studies used the same underlying datasets to look at multiple cancers for example Cai et al. [21].

### 3.2 Literature Review Quality Assessment

Overall, the cohort studies were broadly well‐conducted, performing strongly in the selection of the non‐exposed cohort, confirming that outcomes were absent at baseline, applying appropriate statistical methods, and adequately controlling for confounding (Appendix 3). However, only half (3/6, 50%) reported fully representative cohorts, and two‐thirds (4/6, 66%) clearly described losses to follow‐up or met the predefined threshold for attrition.

Among the case‐control studies, most studies had representative cases (i.e., minimal risk of selection bias) (23/27, 85%), an adequate case definition (26/27, 96%), an adequate definition for the controls (25/27, 93%), appropriate ascertainment of the exposure (24/27, 89%), used the same method to assess the exposure in both groups (26/27, 96%), appropriate statistical methods (25/27, 93%), and accounted for confounding (23/27, 85%) (Appendix 4). Eight (30%) received lower scores for simultaneous ascertainment of exposure and outcome including one (1/8, 13%) that measured the exposure after the outcome.

### 3.3 Literature Review Strength of Evidence

In total, 30 hypotheses were identified in the literature review and evaluated using the GRADE framework (Appendix 5). Two studies that reported on NHL specified analyses on chronic lymphocytic leukaemia (CLL), prolymphocytic leukaemia (PLL), small lymphocytic lymphoma (SLL) subtypes [22, 23]. As the authors classified these as subsets of NHL (rather than leukaemia), they were treated as NHL throughout this analysis.

Among the EBV infection hypotheses, *low certainty* evidence was identified for two (2/13, 15%)‐ cervical and ovarian cancers. Cervical cancer was rated as ‘not serious’ across all relevant domains. Ovarian cancer was not taken further within the review because its effect estimates suggested a protective association. All remaining EBV infection hypotheses had *very low certainty* evidence. One leukaemia study showed inconsistent findings by age group, with older participants showing a possible protective association and younger participants a potential increased risk [24].

Among the IM‐cancer hypotheses, *low certainty* evidence was observed for NHL, leukaemia, male genital, multiple myeloma (MM), prostate and testicular (6/17, 35%). Prostate, MM and NHL were initially downgraded to very low, then upgraded due to large effect sizes. Leukaemia and testicular cancers were rated as ‘not serious’ across all relevant domains. The male genital hypothesis was not taken further in the review due to neutral effect estimate from a single study. The rest of the hypotheses had *very low certainty* evidence.

Across both EBV infection and IM, 13 hypothesis (43%) were advanced to the next stage. Six (6/13, 46%) were advanced because they were rated low certainty (EBV infection‐cervical, IM‐NHL, IM‐leukaemia, IM‐MM, IM‐prostate and IM‐testicular). Three (23%) EBV infection hypotheses were advanced because their corresponding IM hypotheses met the low certainty threshold (EBV infection‐leukaemia, EBV infection‐NHL, EBV infection‐testicular). One (8%) IM hypothesis (IM‐female genital) was advanced as the corresponding EBV infection‐cervical hypothesis was rated low. The IM‐leukaemia finding also supported inclusion of the broader ‘IM‐other haematologic’ category (1/13, 8%), which was later subdivided by haematologic subtype. EBV infection‐CLL/PLL/SLL, EBV infection‐diffuse large B cell lymphoma (DLBCL), or EBV infection‐follicular lymphoma (FL) were not advanced individually because doing so would move from a broader to a more specific classification of NHL. Nonetheless, global burden data are later presented by NHL subtype.

Hypotheses advanced to the next stage had to demonstrate that EBV infection or IM acted as a risk factor, not a protective or neutral exposure.

Finally, we reinstated the EBV infection‐ and IM‐breast hypotheses (2/13, 15%) for advancement, given the results of the prior systematic review that we used as a framework to guide the biological plausibility assessment [13].

### 3.4 Biological Plausibility

The results of the biological plausibility assessment are summarised in Appendix 6. No eligible studies were identified for MM. Leukaemia and other haematologic malignancies were assessed together as a single group.

EBV/EBV‐IM‐breast cancer had a large volume of contributing papers (23), only one (4%) of which contained solely samples that tested negative. Amongst the remaining 22, three (14%) reported results for PCR plus ISH evidence plus/minus IHC data. A further two (9%) reported results for ISH evidence without PCR. The majority of the remaining studies reported results for PCR evidence only.

EBV/EBV‐IM‐NHL studies likely represent a broad array of NHL subtypes. Of the six contributing studies, one (17%) contained solely samples that tested negative. That study only tested 14 samples. Restricting to the five remaining studies, two (40%) reported results for PCR plus ISH plus/minus IHC evidence, and two (40%) others ISH evidence plus/minus IHC evidence.

Of the two EBV/EBV‐IM‐testicular cancer studies, one (50%)‐ which undertook PCR, ISH and IHC testing‐reported Epstein‐Barr Virus‐Encoded Small RNAs (EBER) detection only in reactive lymphocytes, not tumour cells. This study was thus essentially disregarded. Although PCR plus ISH plus IHC evidence was reported in the second and samples tested positive, should be treated with caution due to the use of an in‐house probe.

Among the six EBV/EBV‐IM‐leukaemia studies, five reported results for PCR evidence only (83%) and one (17%) ISH evidence only. All studies contained positive results. EBV/EBV‐IM‐prostate and cervical studies were limited by only PCR evidence being reported, with only three studies across the two cancers.

### 3.5 PAFs/EFs

Data were available for PAF calculations for 11 hypotheses (Table 5). The high global prevalence of EBV infection resulted in comparatively large PAFs relative to IM, as IM does not occur in everyone who becomes infected with EBV. When interpreting IM‐related PAFs, it is important to consider potential information bias in IM studies compared with EBV infection studies, especially when hospitalisation data were used to define the exposure as most IM is managed in outpatient settings. Such under‐ascertainment of IM would likely bias PAF estimates downward, both by reducing the measured prevalence of IM exposure and by misclassifying some exposed individuals as unexposed. Conversely, if cancer risk is more strongly associated with clinically severe IM, hospital‐based definitions may preferentially capture the subgroup most relevant to risk. Evidence remains limited, however, on whether IM severity modifies cancer risk, and hospitalisation is an imperfect proxy that varies across settings. Accordingly, PAF estimates for IM should be interpreted cautiously and are context‐dependent. Among EBV infection hypotheses, NHL and testicular had the highest PAFs, supported by multiple studies. For IM, breast cancer had the lowest value (0.02%) and NHL the highest, again across multiple studies.

**TABLE 5:** PAFs and EFs.

Exposure	Cancer	Country	Age of study population	Antibody/antigen combination	Percentage of people with the cancer of interest who were documented as infected with EBV/had IM (%)	OR/RR/HR	PAF (%)	EF (%)	Study
EBV infection	Cervical	Finland	15 years or older	IgG VCA	69.0	1.4	19.7	28.6	[25]
	Leukaemia	Germany	6 months‐15 years	IgG VCA/EBNA	52.5	2.05	26.9	51.2	[24]
	NHL	USA	30–84 years	IgG VCA	93.8	1.22	16.9	18.0	[22]
		USA	47–95 years	IgG VCA/EBNA‐1/EA‐D/ZEBRA	94.2	1.28	20.6	21.9	[26]
		Czech Republic, France Germany, Ireland, Italy, Spain	Adults < 38 years to ≥ 72 years	IgG VCA/EBNA‐1, IgM VCA	26.3^a^	2.88	17.2	65.3	[27]
					24.2^a^	1.44	7.4	30.6	[27]
		Greece	0–14 years	IgG VCA	76.7	6.73	65.3	85.1	[28]
		Italy	35–65 years	IgG EBNA	86.8	1.4	24.8	28.6	[29]
	Testicular	Norway	Adults; mean 35.7 years	IgG VCA	97.5	2.74	61.9	63.5	[30]
				IgG EBNA (EBNA‐1)	95.1	2.04	48.5	51.0	[30]
IM	Breast	UK (Oxford)	All ages	N/A	0.2	1.14	0.02	12.3	[31]
		UK (England)	All ages	N/A	0.1	1.35	0.02	25.9	[31]
	Leukaemia	UK (England)	All ages	N/A	0.4	2.23	0.24	55.2	[31]
	MM	UK (England)	All ages	N/A	0.2	3.99	0.16	74.9	[31]
	NHL	USA	18–100 years	N/A	0.1	2.7	0.07	63.0	[32]
		UK (Oxford)	All ages	N/A	0.4	1.78	0.19	43.8	[31]
		UK (England)	All ages	N/A	0.7	5.59	0.58	82.1	[31]
	Other haematologic	Denmark	All ages	N/A	0.4	1.61	0.15	37.9	[21]
	Prostate	UK (England)	All ages	N/A	0.1	4.94	0.10	79.8	[31]
	Testicular	UK (Oxford)	All ages	N/A	1.2	1.55	0.43	35.5	[31]

*Note:* PAFs/EFs were calculated per study. To be eligible for PAFs/EFs to be calculated, the study specific effect estimates (OR/RR/HR) had to be adjusted for at least age and sex. The percentage of people with the cancer of interest who were documented as infected with EBV/had IM was also taken from each study. Studies of EBV infection had to have measured this using either IgG‐VCA or IgG‐EBNA. PAFs were calculated using Miettinen/Flegal's formula from advanced hypotheses. Depending on the study design, ages may relate to age at enrolment or age at cancer diagnosis. They may also represent the age range for an underlying study from which the documented study was sampled, as opposed to for the documented study itself. In some instances, the full range of ages was not clear.

Abbreviations: EA, early antigen; EBNA, EBV nuclear antigen; EF, etiologic fraction; HR, hazard ratio; IgG, immunoglobulin G; MM, multiple myeloma; NHL, non‐Hodgkin lymphoma; OR, odds ratio; PAF, population attributable fraction; RR, risk ratio; VCA, viral capsid antigen; ZEBRA, BamHI Z EBV replication activator.

^a^ Two separate subtypes of NHL.

EFs ranged between 12.3% (IM‐breast cancer) and 85.1% (EBV infection‐NHL). Where cancers were represented for both EBV infection and IM, EF results were generally consistent across exposures, in contrast to PAFs, for which differences reflected the large disparity in exposure prevalence.

These PAF and EF estimates are study‐specific. As EBV infection is nearly universal, even small effect estimates yield large PAFs. The very high seroprevalence among cancer cases (e.g., > 90% for testicular cancer) likely reflects background infection rates rather than true etiologic attribution.

### 3.6 GLOBOCAN and IHME

**FIGURE 2:**
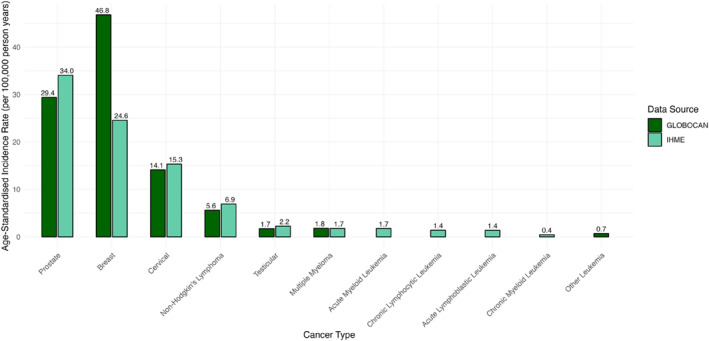
Global age‐standardised incidence rates of the cancers of interest. Age‐standardised incidence rates per 100,000 person years from GLOBOCAN (2022, light green) and IHME (2021, dark green) for the cancers of interest. Numbers at the top of the columns indicate the precise numbers. Note that the GLOBOCAN breast cancer data are for females only, IHME data are for males and females. Prostate, cervical and testicular estimates are single‐sex in both instances. GLOBOCAN, Global Cancer Observatory; IHME, Institute for Health Metrics and Evaluation.

Among the cancers of interest, global age‐standardised incidence rates were highest for breast and prostate cancer (Figure 2). According to IHME estimates, the age‐standardised incidence rates were 46.8 per 100,000 person years for breast cancer and 29.4 per 100,000 person years for prostate cancer. GLOBOCAN reported corresponding rates of 24.6 (due to estimates being for both males and females) and 34.0 per 100,000 person years, respectively.

For the specific leukaemia categories, data were available only from IHME. Both IHME and GLOBOCAN reported separate estimates for MM. IHME also provided data for the CLL subtype of NHL, but not the PLL and SLL subtypes.

There was substantial country‐by‐country variation in the age‐standardised incidence rates of the cancers (Appendix 7). No data were available for MM.

### 3.7 Length of Time Until Onset

A single study reported data on latency between IM and the diagnosis of the cancer of interest [31]. Using hospitalisation data from England, UK, the study found that 10/12 (83%) of NHL was diagnosed within 1 year of hospital admission for IM, which by common practise in cancer epidemiology is excluded due to the potential for reverse causality.

### 3.8 Biomarkers

One study assessed whether biomarkers could predict our cancers of interest [33]. Among men 40–59 years of age, higher midlife prostate‐specific antigen (PSA) levels were strongly predictive of increased long‐term risk of prostate cancer (AUC 0.75–0.83, depending upon age and whether the cancer was lethal).

## 4 Discussion

We conducted a literature review with accompanying quality assessment, GRADE, and biological plausibility assessments to evaluate cancers potentially associated with EBV infection and IM beyond BL, HL, GC and NPC. For cancers advanced on their GRADE results, we summarised available data on disease burden, timing onset after IM, PAFs, EFs, and biomarkers.

Table 6 summarises findings for the eight cancers advanced for further evaluation (breast, cervical, leukaemia, MM, NHL, other haematologic, prostate, and testicular) grouped into six broader cancer categories. Across these categories, NHL, MM and prostate cancer demonstrated the largest EFs (either as standalone estimates or within a range), while NHL and testicular cancer showed the largest PAFs within their respective grouping (i.e., EBV infection or IM). Breast cancer and NHL had the greatest weight of evidence for biological plausibility, cervical and prostate cancers the least. Despite using an array of tests, the testicular cancer studies were limited in their evidence. Only prostate cancer had an identified predictive biomarker, and only NHL had information on the timing of onset following IM. The highest global burdens were observed for breast, cervical and prostate cancers.

**TABLE 6:** Summary table of advanced exposure‐outcome hypotheses.

Exposure‐outcome hypothesis	GRADE rating	PAF/EFs calculable?	Burden estimates found?	Biomarkers found?	Timing data found?
EBV infection associated with:					
Breast	Very low	No	Yes	No	N/A
Cervical	Low	Yes	Yes	No	N/A
Leukaemia	Very low	Yes	Yes	No	N/A
NHL	Very low	Yes	Yes	No	N/A
Testicular	Very low	Yes	Yes	Yes	N/A
IM associated with:					
Breast	Very low	Yes	Yes	No	No
Female genital	Very low	No	Yes	No	No
Leukaemia	Low	Yes	Yes^a^	No	No
MM	Low	Yes	Yes	No	No
NHL	Low	Yes	Yes	No	Yes
Other haematologic	Very low	Yes	Yes^b^	No	No
Prostate	Low	Yes	Yes	Yes^c^	No
Testicular	Low	Yes	Yes	No	No

*Note:* Summary of findings for the advanced hypotheses, including data availability. Burden estimates, and biomarkers assessed for the cancer, not for the hypothesis.

Abbreviations: EBV, Epstein‐Barr virus; EF, etiologic fraction; IM, infectious mononucleosis; MM, multiple myeloma; NHL, non‐Hodgkin lymphoma; PAF, population attributable fraction.

^a^ The burden of leukaemia was assessed in subtypes where possible in IHME and GLOBOCAN.

^b^ Although no overall burden estimate was available for other haematologic cancers, this was noted as ‘yes’ due to the availability of relevant subtype data.

^c^ Although no IM‐specific biomarkers were identified, biomarkers of EBV infection were considered relevant and are included here.

Our findings complement and extend evidence from cancers with established EBV associations, recognising that the proof of such causal links often emerged over decades. For example, in the case of BL in the 1960s, the presence of replication‐competent EBV in tumour cells provided strong biological plausibility, although the exact pathogenic mechanism remains uncertain [34]. The most compelling evidence to date for an EBV‐MS association came from a large, long‐term epidemiological study in US Army recruits, published in 2022 [5]. This landmark study paralleled the design and rigour of the largest cohort studies identified within our review, and followed preceding epidemiological studies and biological experiments for example [35, 36]. Again, the exact mechanism of EBV action is subject to debate [34]. Finally, large pooled analyses from international collaborations, such as the InterLymph consortium, have also contributed substantially to understanding lymphoma risk factors, including infectious exposures such as EBV [37].

Strengths of this work include its staged approach integrating multiple complementary assessments (i.e., literature synthesis, quality appraisal, GRADE evaluation, and biological plausibility assessment) to triangulate evidence on EBV‐associated cancers. This multidimensional approach allowed for additional context to characterise the strength and coherence of evidence across diverse study designs. The use of predefined GRADE criteria and an explicit set of modifications to the Newcastle‐Ottawa Scale enhanced transparency and reproducibility. By combining epidemiologic and biological perspectives, the review also provides a foundation for prioritising hypotheses with the greatest potential biological relevance, which can inform future mechanistic and observational work.

Limitations include single‐reviewer screening, limited inclusion of non‐English databases, and heterogeneity in study quality and generalisability. The GRADE process, though structured and transparent, involves some subjective interpretation in how evidence is pooled and hypotheses are grouped. For example, the large effect sizes reported by Goldacre et al. [31] for IM‐pancreatic cancer was subsumed under the broader digestive cancer hypothesis. Similarly, effect sizes for IM‐oral cavity, pharynx, and lip cancers were not upgraded due to inconsistency across studies, and IM‐malignant brain cancer results were too borderline to justify upgrading. For Akre et al. [30], heterogeneity in antibody‐specific effects prevented upgrading of the EBV infection–testicular cancer hypothesis. Extensive between‐study variability precluded formal meta‐analysis. We were also limited by a lack of studies on biomarkers and latency intervals. We note potential information bias due to inclusion of IM cases without EBV confirmation, which could represent alternative aetiologies. Finally, despite a rigorous and comprehensive search strategy, studies in which infectious mononucleosis or EBV‐related exposures were not explicitly referenced in titles or abstracts may not have been identified during screening, particularly when these exposures were assessed alongside broader childhood, social, or environmental factors.

Our research indicates that eight additional cancers (across six groups) warrant further investigation to clarify whether, and through what mechanisms, EBV may contribute to their development. These findings suggest that EBV‐associated malignancy may represent a broader public health burden than previously appreciated. While prophylactic vaccination remains a key area of interest, improved understanding of EBV's role in malignancy may also inform therapeutic or adjunctive strategies, including therapeutic vaccination, antiviral approaches, and immunomodulatory interventions. This may be particularly relevant for cancers with long latency or indirect viral involvement.

Additionally, future research evaluating the link between EBV/EBV‐IM and potentially related cancers should leverage large, nationally representative datasets and fit‐for‐purpose observational study designs. A major gap in the evidence base is the lack of longitudinal, biomarker‐based studies of EBV seroconversion and subsequent infectious mononucleosis during adolescence, as such studies have largely focused on young adults or early childhood, despite adolescence being a critical risk period [38, 39, 40, 41, 42]. Addressing this gap will require studies designed to accommodate long latency, low incidence, and secular trends in cancer epidemiology. These studies should account for long latency, low incidence and secular trends of cancer epidemiology. Designs such as case‐cohort or nested case‐control studies, target trial emulation using routinely collected data, and quasi‐experimental approaches (e.g., interrupted time series [43] or difference‐in‐differences analysis [44]) can strengthen causal inference. Increasingly, linked record systems, such as the Clinical Practice Research Datalink (CPRD) [45] in the UK and Scandinavian national registries, enable long‐term follow‐up, outcome validation‐ and through tokenised linkages‐the ability to combine randomised trial data with real‐world evidence [46]. Complementary laboratory studies, with careful assay selection to ensure robust methods of viral detection, could further support mechanistic understanding.

When randomised controlled trials are infeasible for outcomes with long latency, convergent evidence from observational, quasi‐experimental, and mechanistic studies is essential to build confidence in the underlying biology and expected impact of EBV vaccines. Such studies could employ target trial emulation frameworks [47] or pragmatic [48] and registry‐based trials [49] embedded in vaccine roll‐outs to evaluate long‐term effects, similar to the approach taken for human papillomavirus vaccines [50]. Non‐specific vaccine effects, such as those proposed by Bacille Calmette‐Guérin vaccine and measles vaccines [51, 52], could merit exploration in the EBV context, potentially revealing broader immunological effects.

## 5 Conclusion

In conclusion, through a comprehensive literature review and a series of structured assessments including study quality, GRADE, and biological plausibility, we identified eight potential additional cancers (within six cancer groups) with a reasonable degree of evidence for potential association with EBV infection or IM. Three cancers (breast, cervical, prostate) showed the highest global burden, with age‐standardised incidence rates exceeding 10 per 100,000 person years. Although specific biomarkers have not yet been established for EBV‐related cancers, examples from other contexts, such as PSA for prostate cancer [53], illustrate how validated biomarkers could help accelerate the evaluation of long term‐outcomes following EBV vaccination. Collectively, these findings expand current understanding of EBV's oncogenic potential beyond the four well‐established malignancies (i.e., BL, HL, GC, and NPC) and highlight the need for multidisciplinary research integrating epidemiologic, clinical and laboratory approaches to clarify mechanisms and inform future prevention strategies.

## Author Contributions

H.R.S., M.D.M., G.S.T. and J.D.D. conceptualised the work. E.S. performed the project administration for the work. M.D.M. curated and analysed the data, the other authors supervised her doing this. M.D.M. and H.R.S. prepared the original draft, the other authors reviewed and edited the work.

## Funding

M.D.M., G.S.T. and H.R.S. declare funding from Moderna Inc., for this work.

## Ethics Statement

The authors have nothing to report.

## Consent

The authors have nothing to report.

## Conflicts of Interest

M.D.M., G.S.T. and H.R.S. declare funding from Moderna Inc., for this work. J.D.D. and E.S. are employees of Moderna and may hold stocks and/or stock options. Outside of this work, G.S.T. has previously received money from Cancer Research UK, Blood Cancer UK, the UK Medical Research Council, Melanoma UK, Pfizer, and the University of Birmingham to support his research team. H.R.S. has previously received grant funding from the Wellcome Trust and Moderna Inc., outside of this work but on the topic of Epstein Barr Virus, and has also undertaken consultancy for Bavarian Nordic.

## Permission to Reproduce Material From Other Sources

The authors have nothing to report.

## Data Availability

The authors have nothing to report.

## Appendix 1 Search Terms

### Embase Classic + Embase <1947 to 2024 July 29>

Detailed search terms and Boolean logic used for MEDLINE, Embase, and Web of Science (to July 2024) to identify studies examining Epstein–Barr virus (EBV) infection, infectious mononucleosis (IM), and malignancy.

**Table:**

Line	Terms	Hits
1	Epstein‐Barr virus.mp. or Epstein barr virus/	68,347
2	Human herpesvirus 4.mp.	170
3	Epstein barr virus/or infectious mononucleosis/or Epstein barr virus infection/	65,366
4	HHV‐4.mp.	96
5	HHV4.mp.	41
6	EBV.mp.	47,507
7	Glandular fever.mp.	451
8	cancer.mp. or malignant neoplasm/	4,885,554
9	Neoplasm/ or neoplasm.mp.	830,922
10	malignan*.mp.	1,273,035
11	Leukaemia/ or leukaemia.mp.	597,060
12	leukaemia.mp.	62,893
13	lymphoma.mp. or lymphoma/	426,015
14	Carcinoma/ or carcinoma.mp.	1,559,724
15	tumour.mp.	404,926
16	tumour.mp.	3,859,126
17	Blastoma/ or blastoma.mp.	4233
18	Melanoma/ or melanoma.mp.	272,659
19	cytoma.mp.	96
20	sarcoma.mp. or sarcoma/	198,492
21	Epidemiology/ or epidemiology.mp.	1,755,384
22	epidemiol*.mp.	2,009,675
23	Case control study.mp. or case control study/	272,910
24	Cohort analysis.mp. or cohort analysis/	1,203,415
25	Longitudinal study.mp. or longitudinal study/	243,479
26	Cross‐sectional study.mp. or cross‐sectional study/	697,938
27	1 or 2 or 3 or 4 or 5 or 6 or 7	86,000
28	8 or 9 or 10 or 11 or 12 or 13 or 14 or 15 or 16 or 17 or 18 or 19 or 20	7,563,896
29	21 or 22 or 23 or 24 or 25 or 26	3,946,636
30	27 and 28 and 29	5355

### Ovid MEDLINE(R) ALL <1946 to July 29, 2024>

**Table:**

Line	Terms	Hits
1	Epstein‐Barr virus.mp. or Epstein barr virus/	45,021
2	Human herpesvirus 4.mp.	905
3	Epstein barr virus/or infectious mononucleosis/ or Epstein barr virus infection/	36,387
4	HHV‐4.mp.	60
5	HHV4.mp.	27
6	EBV.mp.	31,745
7	Glandular fever.mp.	278
8	cancer.mp. or malignant neoplasm/	2,547,854
9	Neoplasm/ or neoplasm.mp.	1,304,156
10	malignan*.mp.	735,571
11	Leukaemia/ or leukaemia.mp.	354,467
12	leukaemia.mp.	39,763
13	lymphoma.mp. or lymphoma/	279,462
14	Carcinoma/ or carcinoma.mp.	1,010,294
15	tumour.mp.	251,323
16	tumour.mp.	2,110,544
17	Blastoma/ or blastoma.mp.	1202
18	Melanoma/ or melanoma.mp.	162,331
19	cytoma.mp.	36
20	sarcoma.mp. or sarcoma/	128,394
21	Epidemiology/ or epidemiology.mp.	2,349,990
22	epidemiol*.mp.	2,514,787
23	Case control study.mp. or case control study/	367,539
24	Cohort analysis.mp. or cohort analysis/	352,663
25	Longitudinal study.mp. or longitudinal study/	200,601
26	Cross‐sectional study.mp. or cross‐sectional study/	592,500
27	1 or 2 or 3 or 4 or 5 or 6 or 7	54,758
28	8 or 9 or 10 or 11 or 12 or 13 or 14 or 15 or 16 or 17 or 18 or 19 or 20	4,811,730
29	21 or 22 or 23 or 24 or 25 or 26	3,443,207
30	27 and 28 and 29	3155

### Web of Science ‐ Web of Science Core Collection to 29^th^ July 2024

**Table:**

Line	Terms	Hits
1	ALL=(Epstein‐Barr virus)	49,767
2	ALL=(Epstein‐Barr virus infections)	21,915
3	ALL=(Herpesvirus 4)	6313
4	ALL=(HHV4)	25
5	ALL=(HHV‐4)	66
6	ALL=(EBV)	33,031
7	ALL=(infectious mononucleosis)	5493
8	ALL=(glandular fever)	254
9	ALL=(Cancer)	4,735,354
10	ALL=(neoplasms)	245,429
11	ALL=(malignan*)	757,049
12	ALL=(leukaemia)	428,707
13	ALL=(leukemia)	460,103
14	ALL=(lymphoma)	311,063
15	ALL=(carcinoma)	1,115,482
16	ALL=(Tumour)	2,313,927
17	ALL=(Tumour)	2,393,620
18	ALL=(Blastoma)	8126
19	ALL=(Melanoma)	212,205
20	ALL=(cytoma)	57
21	ALL=(sarcoma)	132,989
22	ALL=(epidemiol*)	1,591,354
23	ALL=(case‐control studies)	166,176
24	ALL=(cohort studies)	867,502
25	ALL=(longitudinal studies)	417,497
26	ALL=(cross‐sectional studies)	548,953
27	#1 OR #2 OR #3 OR #4 OR #5 OR #6 OR #7 OR #8	66,213
28	#9 OR #10 OR #11 OR #12 OR #13 OR #14 OR #15 OR #16 OR #17 OR #18 OR #19 OR #20 OR #21	6,439,447
29	#22 OR #23 OR #24 OR #25 OR #26	3,052,632
30	#27 AND #28 AND #29	4239

## Appendix 2 Included Studies

Summary of all studies meeting the inclusion criteria, including design, population, exposure definition, cancer type, data source, and effect estimates. All strength of association results used seronegative as the baseline.

**Table:**

First author	Study design	Years of study	Country/ies of study	Population	Sample size	Exposure definition (antibody/antigen combination to test for EBV infection, DNA to indicate EBV infection, or IM)	Type of cancer(s)	Source of outcome data	Strength of association (CI in brackets)
Agborsangaya [54]	Case‐control (nested)	1983‐?	Finland	Pregnant females	108 cases, 208 controls	IgG EBNA‐1, EBNA‐1/ZEBRA	Pregnancy associated breast	Cancer registry	EBNA‐1 aOR 0.7 (0.2–2.2) EBNA‐1/ZEBRA aOR 1.7 (1.0–2.8)
Akre [30]	Case‐control (nested)	1973–1993	Norway	Males	81 cases, 242 controls	IgG EBNA, EBNA‐1, VCA	Testicular	Cancer registry	EBNA aOR 2.04 (0.59–7.01) EBNA‐1 aOR 2.04 (0.59–7.01) VCA aOR 2.74 (0.62–12.12)
Bertrand [22]	Case‐control (nested)	1982–2003	USA	Male physicians and female nurses	340 cases, 662 controls	IgG VCA	NHL (CLL/SLL)	Medical records, histologically confirmed	VCA aOR 1.22 (0.71–2.12)
Cai [21]	Cohort	1973–2016	Denmark	General population	1,419,407	EBV‐IM	Multiple cancers	Cancer registry	IM other (non‐lymphoid) haematologic aHR 1.61 (0.23–11.51) IM other (non‐haematologic) aHR 1.41 (1.01–2.00)
Coghill [55]	Case‐control (nested)	1998	Norway/USA	European ancestry cohorts	360 cases, 397 controls	Any antibody VCA, EBNA‐1	Glioma (including subtypes)	Histological criteria	Norway VCA aOR 0.55 (0.32–0.94) Norway EBNA‐1 aOR 0.97 (0.60–1.58) USA VCA aOR 0.34 (0.08–1.47) USA EBNA‐1 aOR 0.46 (0.14–1.55)
Cox [19]	Case‐control (nested)	1973–2003	Norway	Females	399 cases, 399 controls	IgG VCA	Breast	Cancer registry	VCA aOR 0.7 (0.1–4.0)
Dagash [20]	Case‐control	2017–2018	Iraq	Hospital patients and healthy neighbour‐hood controls	123 cases, 123 controls	IgM (no specific antigen)	Malignant neck mass	Medical records, diagnostic criteria	aOR 1.37 (0.56–3.38)
De Roos [23]	Case‐control (nested)	1994–2009	USA	Community dwelling, post‐menopausal, women	491 cases, 491 controls	IgG VCA, EBNA‐1	NHL (CLL/PLL/SLL)	Medical records	VCA aOR 0.9 (0.5–1.3) EBNA‐1 aOR 0.5 (0.3–0.8)
de Sanjose [27]	Case‐control	1998–2003	Czech Republic, France Germany, Ireland, Italy, Spain	European lymphoma patients with population or non‐cancer hospitalised population controls	1085 cases, 1153 controls	IgG VCA/EBNA‐1 IgM VCA	B‐cell, T‐cell (including subtypes, not including HL)	Medical records, 20% of samples independently histologically confirmed.	B‐cell aOR 2.88 (2.16–3.85) T‐cell aOR 1.44 (0.88–2.37)
Dillner [56]	Case‐control	1984–1991	Sweden	Females admitted for treatment and population‐based controls	94 cases, 188 controls	IgG EBNA‐1	Cervical	Medical records	EBNA‐1 aOR 0.97 (0.53–1.78)
Goldacre [31]	Cohort	1963–2005	UK	Individuals admitted to hospital	17,826 IM, 3,949,220 no IM	IM	Multiple cancers	Medical records	ENGLAND Oral cavity, pharynx, lip aHR 5.54 (1.14–16.2) Colon aHR 1.19 (0.25–3.48) Pancreas aHR 7.73 (1.59–22.7) Breast aHR 1.35 (0.37–3.45) Prostate aHR 4.94 (1.02–14.5) All skin aHR 0.72 (0.15–2.09) Malignant brain aHR 2.15 (0.58–5.52) NHL aHR 5.59 (2.88–9.79) MM aHR 3.99 (0.10–22.3) Leukaemia aHR 2.23 (0.72–5.23) OXFORD REGION Rectum aHR 1.39 (0.17–5.04) Colon aHR 0.41 (0.01–2.28) Breast aHR 1.14 (0.42–2.48) Lung aHR 0.30 (0.01–1.70) Testis aHR 1.55 (0.19–5.69) Bladder aHR 1.74 (0.36–5.10) All skin aHR 1.05 (0.22–3.07) NHL aHR 1.78 (0.37–5.23)
Hakama [25]	Case‐control	1962–1981	Finland	Females, general population cases and controls	32 cases, 64 controls	IgG VCA	Cervical	Cancer registry	VCA aOR 1.4 (0.5–3.8)
Heng [57]	Case‐control	2012–2016	Canada/USA	Adult female participants in web‐based surveys of breast cancer risk factors	3497 IM, 12,739 no IM	IM	Invasive breast	Self‐report	aOR 0.83 (0.72–0.94) (most adjusted model)
Hjalgrim [58]	Cohort	1960s‐1995	Denmark, Sweden	General population	38,562 IM, standardised	EBV‐IM	Multiple cancers	Cancer registry	Buccal cavity and pharynx aSIR 0.73 (0.47–1.09) Digestive organs and peritoneum aSIR 0.91 (0.79–1.05) Respiratory organs aSIR 0.75 (0.62–0.89) Breast aSIR 1.07 (0.92–1.23) Female genital organs aSIR 1.05 (0.88–1.25) Male genital organs aSIR 0.96 (0.76–1.19) Urinary system aSIR 0.81 (0.63–1.01) Skin aSIR 1.27 (1.13–1.43) Other specified sites aSIR 1.11 (0.92–1.34) NHL aSIR 1.21 (0.87–1.64) MM aSIR 1.19 (0.61–2.08) Leukaemia aSIR 1.26 (0.90–1.71)
Kartsonaki [59]	Case‐control (nested)	2004–2016	China	General population	597 cases, 1986 controls	VCA, EBNA‐1, EA‐D, ZEBRA (no specific antibody)	Oesophageal, gastric and duodenal cancers	Medical records	VCA aHR 0.59 (0.04–8.96)
Koshiol [32]	Cohort	1969–1996	USA	Male veterans (black or White)	4,501,578	IM	NHL	Medical records	aHR 2.7 (1.4–5.5)
Lehtinen [60]	Case‐control (nested)	1968–1980s	Finland	General population	11 cases, 22 controls	IgG VCA, EBNA	NHL	Cancer registry	VCA aOR 0.6 (0.1–4.3) EBNA aOR 0.4 (0.1–2.0)
Mahjour [61]	Case‐control	2007–2008	Iran	Children admitted to hospital and children attending a routine appointment	90 cases, 90 controls	IgG VCA, EBNA‐1	ALL	Medical records	VCA OR 2.7 (1.28–5.58) EBNA‐1 OR 0.84 (0.47–1.50)
Massa [62]	Cohort	1989–2007	USA	Female nurses	81,807	IM	Invasive breast	Self‐report, medical records	aHR 1.00 (0.90–1.11)
Michos [28]	Case‐control	1996–2003	Greece	Newly diagnosed children and children attending the hospital for a minor ailment	73 cases, 73 controls	IgG VCA	NHL	Medical records, histologically confirmed	VCA aOR 6.73 (1.45–31.20)
Richardson [63]	Case‐control	1992–1995	Australia	Females under 40 newly diagnosed and population controls	208 cases, 169 controls	IgG VCA	Breast	Cancer registry	VCA aOR 1.11 (0.34–3.73)
Richiardi [29]	Case‐control (nested)	1993–2005	Italy	Adults taking part in a study on cancer and diet	91 cases, 182 controls	IgG EBNA	NHL	Cancer registry, mortality registry, local demographic offices, medical records, active follow‐up	aOR 1.4 (0.7–2.8)
Roderburg [64]	Cohort	2000–2018	Germany	> 14 years olds registered with primary care	12,095 IM, 12,095 no IM	IM	Multiple cancers	Medical records	Prostate aHR 3.09 (1.23–7.76) Lip, oral cavity and pharynx aHR 1.48 (0.49–5.53) Lymphoid and other haematologic aHR 1.75 (1.22–2.50) Female genital aHR 1.46 (0.40–5.53) Breast aHR 1.21 (0.80–1.85) Skin aHR 1.20 (0.85–1.71) Urinary tract aHR 1.15 (0.56–2.36) Digestive aHR 0.80 (0.51–1.26) Respiratory aHR 0.57 (0.29–1.09)
Sawaya [65]	Case‐control	2012–2014	France	Newly diagnosed males and population controls	803 cases, 866 controls	IM	Prostate	Medical records, histologically confirmed	aOR 0.92 (0.46–1.86)
Schlehofer [24]	Case‐control	1990–1991	Germany	Children 6 months‐15 years and controls treated for other conditions	104 cases, 168 controls	IgG VCA/EBNA	Leukaemia	Cancer registry	0–5 year‐olds aOR 2.05 (0.99–4.23) 6+ year‐olds aOR 0.48 (0.19–1.21)
Sjostrom [66]	Case‐control (nested)	1990s–2000s	Denmark, Sweden	Adults taking part in a series of underlying studies	197 cases, 394 controls	IgG EBNA‐1	Glioma	Cancer registry	EBNA‐1 aOR 0.64 (0.39–1.06)
Steininger [67]	Case‐control	2004–2005	Austria and USA	Cases from Austrian/USA cohorts and White controls presenting for testing of immunity post‐vaccination from Austria	200 cases, 100 controls	IgG (no specific antigen)	CLL	Unknown	OR 0.43 (0.17–1.07)
Teras [26]	Case‐control (nested)	1998–2007	USA	Adults in a nutrition cohort	225 cases, 449 controls	IgG VCA/EBNA‐1/EA‐D/ZEBRA	NHL	Medical records, cancer registry using ICD‐10 codes and WHO classifications	Two or more antigens aOR 1.28 (0.67–2.47)
Trabert [68]	Case‐control (nested)	1993–2010	Poland/USA	Females newly diagnosed and population control/controls from control arm of a screening trial	404 cases and 715 controls	Any antibody EBNA‐1/EA‐D/ZEBRA	Ovarian	Medical records, cancer registry	Poland aOR 0.86 (0.43–1.75) USA aOR 0.90 (0.33–2.44)
Wang [69]	Case‐control	1998–2002	USA	Twin study	162 twin pairs	IM	NHL	Self‐report	aOR 0.35 (0.14–0.90)
Wrensch [70]	Case‐control	1991–1995	USA	Newly diagnosed adults and population controls	134 cases, 165 controls	IgG (no specific antigen)	Glioma	Medical records	aOR 0.6 (0.3–1.4)
Wrensch [71]	Case‐control	1997–1999	USA	Newly diagnosed adults and population controls	229 cases, 289 controls	IgG VCA	Glioma	Medical records	VCA aOR 0.82 (0.40–1.67)
Zhang [72]	Case‐control	2008–2012	China	Females with newly diagnosed cancer and females attending health check‐ups	671 cases, 859 controls	DNA	Breast	Medical records, histologically confirmed	DNA aOR 1.05 (0.81–1.37)

Abbreviations: ALL, acute lymphoblastic leukaemia; a, effect estimate controlling for confounding by age and sex either through matching, restriction of the participants, or adjustment; CI, 95% confidence interval; CLL, chronic lymphocytic leukaemia; EA‐D, early antigen diffuse; EBNA, EBV nuclear antigen; EBV, Epstein‐Barr virus; HL, Hodgkin lymphoma; HR, hazard (i.e., rate) ratio; IgG, immunoglobulin G; IM, infectious mononucleosis; MM, multiple myeloma; NHL, non‐Hodgkin lymphoma; OR, odds ratio; PLL, prolymphocytic leukaemia; RR, risk ratio; SIR, standardised incidence ratio; SLL, small lymphocytic lymphoma; VCA, viral capsid antigen; ZEBRA, BamHI Z EBV replication activator.

## Appendix 3 Quality Assessment for the Cohort Studies

Modified Newcastle–Ottawa Scale scores for cohort studies included in the review, using criteria adapted from Deeks et al. [12].

**Table:**

First author	Representativeness of the exposed cohort (1)	Selection of the non‐exposed cohort (1)	Ascertainment of the exposure (1)	Outcome not present at start (1)	Comparability of cohorts (2)	Assessment of outcome (1)	Follow‐up long enough (1)	Adequacy of follow‐up (1)	Appropriate statistical methods (1)	Control of confounders (1)
Cai [21]	1	1	1	1	2	1	1	1	1	1
Goldacre [31]	1	1	1	1	1	1	U	U	1	1
Hjalgrim [58]	1	1	1	1	1	1	1	1	1	1
Koshiol [32]	0	1	1	1	U	1	1	U	1	1
Massa [62]	0	1	0	1	1	1	1	0	1	1
Roderburg [64]	0	1	1	1	1	1	1	0	1	1

Abbreviation: U, unknown.

## Appendix 4 Quality Assessment for the Case‐Control Studies

Modified Newcastle–Ottawa Scale scores for case–control studies included in the review, using criteria adapted from Deeks et al. [12].

**Table:**

First author	Adequacy of case definition (1)	Representativeness of the cases (1)	Selection of controls (1)	Definition of controls (1)	Comparability of cases and controls (2)	Ascertainment of exposure (1)	Same method for cases and controls (1)	Non‐response rate (1)	Appropriate statistical methods (1)	Control of confounders (1)	Simultaneous ascertainment of exposure and outcome (−1)
Agborsangaya [54]	1	1	1	1	2	1	1	1	1	1	0
Akre [30]	1	1	0	1	1	1	1	1	1	1	0
Bertrand [22]	1	1	1	1	2	1	1	1	1	1	0
Coghill [55]	1	1	1	1	2	1	1	1	1	1	0
Cox [19]	1	1	1	1	2	1	1	1	1	1	0
Dagash [20]	1	1	1	1	2	1	1	U	1	0	−1
De Roos [23]	1	1	1	1	2	1	1	1	1	1	0
de Sanjose [27]	1	1	0	1	2	1	1	U	1	1	0
Dillner [56]	1	1	1	U	1	1	1	U	1	1	0
Hakama [25]	1	1	1	1	2	1	U	U	1	1	0
Heng [57]	0	0	0	0	0	0	1	0	1	1	−1
Kartsonaki [59]	1	1	1	1	0	1	1	1	1	1	0
Lehtinen [60]	1	1	1	1	2	1	1	1	1	1	0
Mahjour [61]	1	1	1	1	1	1	1	1	0	0	−1
Michos [28]	1	1	0	1	1	1	1	0	1	1	−1
Richardson [63]	1	1	1	1	1	1	1	1	1	1	0
Richiardi [29]	1	1	1	1	2	1	1	1	1	1	0
Sawaya [65]	1	1	1	1	1	0	1	U	1	1	0
Schlehofer [24]	1	1	0	1	1	1	1	0	1	1	−1
Sjostrom [66]	1	0	1	1	2	1	1	1	1	1	0
Steininger [67]	1	1	0	1	1	1	1	U	0	0	−1
Teras [26]	1	0	1	1	2	1	1	1	1	1	0
Trabert [68]	1	1	1	1	1	1	1	1	1	1	0
Wang [69]	1	0	1	1	1	0	1	U	1	1	0
Wrensch [70]	1	1	0	1	2	1	1	1	1	0	−1
Wrensch [71]	1	1	1	1	2	1	1	1	1	1	0
Zhang [72]	1	1	0	1	1	1	1	U	1	1	−1

Abbreviation: U, unknown.

## Appendix 5 Results from the GRADE Assessment

Results presented per hypothesis across the GRADE domains, including upgrading and downgrading criteria. Risk of bias, inconsistency, indirectness and imprecision were rated either very serious, serious, or not serious.

**Table:**

Hypothesis	Risk of bias	Inconsistency	Indirectness	Imprecision	Large effect	Dose response	Confounding	Overall rating	No. of studies	Study
EBV INFECTION										
ALL	Serious	Serious	Not serious	Not serious	0	0	0	Very low	1	[61]
Breast	Serious	Serious	Not serious	Not serious	0	0	0	Very low	4	[19, 54, 63, 72]
Cervical	Not serious	Not serious	Not serious	Not serious	0	0	0	Low	2	[25, 56]
CLL/PLL/SLL	Serious	Very serious	Very serious	Not serious	0	0	0	Very low	3	[22, 23, 67]
DLBCL	Not serious	Very serious	Very serious	Not serious	0	0	0	Very low	2	[22, 23]
FL	Not serious	Serious	Very serious	Not serious	0	0	0	Very low	2	[22, 23]
Glioma	Serious	Not serious	Not serious	Not serious	0	0	0	Very low	4	[55, 66, 70, 71]
Leukaemia	Serious	Serious	Not serious	Not serious	0	0	0	Very low	1	[24]
Malignant neck mass	Serious	Not serious	Not serious	Not serious	0	0	0	Very low	1	[20]
NHL	Serious	Very serious	Serious	Not serious	0	0	0	Very low	7	[22, 23, 26, 27, 28, 29, 60]
Oesophageal	Serious	Not serious	Not serious	Serious	0	0	0	Very low	1	[59]
Ovarian	Not serious	Not serious	Not serious	Not serious	0	0	0	Low	1	[68]
Testicular	Serious	Not serious	Not serious	Not serious	0	0	0	Very low	1	[30]
IM										
Breast	Serious	Serious	Serious	Not serious	0	0	0	Very low	5	[31, 57, 58, 62, 64]
Digestive	Not serious	Serious	Serious	Not serious	0	0	0	Very low	3	[31, 58, 64]
Female genital	Not serious	Not serious	Serious	Not serious	0	0	0	Very low	2	[58, 64]
Leukaemia	Not serious	Not serious	Not serious	Not serious	1	0	0	Low	2	[31, 58]
Lip/oral cavity/pharynx/buccal	Not serious	Not serious	Serious	Not serious	0	0	0	Very low	3	[31, 58, 64]
Lymphoid	Not serious	Not serious	Serious	Not serious	0	0	0	Very low	1	[64]
Male genital	Not serious	Not serious	Not serious	Not serious	0	0	0	Low	1	[58]
Malignant brain	Not serious	Not serious	Not serious	Serious	0	0	0	Very low	1	[31]
MM	Not serious	Not serious	Not serious	Serious	1	0	0	Low	2	[31, 58]
NHL	Not serious	Not serious	Very serious	Not serious	1	0	0	Low	4	[31, 32, 58, 69]
Other haematologic	Not serious	Not serious	Not serious	Serious	0	0	0	Very low	1	[21]
Prostate	Not serious	Not serious	Very serious	Not serious	1	0	0	Low	3	[31, 64, 65]
Rectum	Not serious	Not serious	Not serious	Serious	0	0	0	Very low	1	[31]
Respiratory	Not serious	Not serious	Serious	Serious	0	0	0	Very low	3	[31, 58, 64]
Skin	Not serious	Not serious	Serious	Not serious	0	0	0	Very low	3	[31, 58, 64]
Testicular	Not serious	Not serious	Not serious	Not serious	0	0	0	Low	1	[31]
Urinary tract	Not serious	Not serious	Serious	Not serious	0	0	0	Very low	3	[31, 58, 64]

Abbreviations: ALL, acute lymphoblastic leukaemia; CLL, chronic lymphocytic leukaemia; DLBCL, diffuse large B cell lymphoma; EBV, Epstein‐Barr virus; FL, follicular lymphoma; IM, infectious mononucleosis; MM, multiple myeloma; NHL, non‐Hodgkin lymphoma; PLL, prolymphocytic leukaemia; SLL, small lymphocytic lymphoma.

## Appendix 6 Biological Plausibility

Summary of molecular studies assessing Epstein–Barr virus (EBV) detection in tumour tissue for advanced hypotheses, including detection method (PCR, ISH, IHC) and results.

**Table:**

Author	Cancer subtype	Where EBV samples were collected	Cases	Controls	Highest number of EBV positive cases by any technique	Was PCR performed?	EBV positivity by PCR	Was EBER ISH performed?	EBV positivity by ISH	Was IHC performed?	EBV positivity by IHC	Markers used to detect EBV by IHC	All EBV detection markers used in study
Breast													
Aboulkassim [73]	Invasive, in situ	Syria	108	0	56 of 108	Yes	56 of 108	No		Yes	Not reported	LMP1	LMP1, EBNA1
Al Hamad [74]	Invasive, in situ	Jordan	100	50	24 of 100	Yes	24 of 100	Yes	Not reported	No			EBNA2, EBER
Antonsson [75]	Invasive, mixed, DCIS	Australia	54	10	5 of 54	Yes	5 of 54	No		No			BALF5
Charostad [76]	Invasive ductal, lobular	Iran	51	51	6 of 51	Yes	6 of 51	No		No			EBNA1
Dowran [77]	Invasive ductal, others	Iran	150	150	0 of 150	Yes	0 of 150	No		No			BHRF1
El‐Naby [78]	Invasive ductal	Egypt	42	42	10 of 42	Yes	12 of 42	No		Yes	10 of 42	LMP1	EBNA1, LMP1
Fessahaye [79]	Invasive ductal, others	Eritrea	144	63	40 of 144	Yes	40 of 144	Yes	5 of 14	Yes	7 of 45	LMP2	EBER, LMP1, LMP2
Glenn [80]	Invasive ductal, in situ	Australia	50	40	34 of 50	Yes	34 of 50	No		Yes	3 of 10	EBNA1, LMP1	EBNA1, LMP1, LMP2
Gupta [81]	Invasive ductal, invasive lobular, mucinous, unknown	Qatar	74	0	36 of 74	Yes	36 of 74	No		No			EBNA1, LMP1
Hussein [82]	Invasive, infiltrative, recurrent	Iraq	22	10	11 of 22	No		Yes	11 of 22	No			EBER
Khabaz [83]	Invasive ductal carcinoma, lobular, medullary, mucinous	Jordan	92	49	24 of 92	Yes	24 of 92	No		Yes	24 of 92	EBNA1	EBER2, EBNA1, EBNA2, LMP1, gp220
Metwally [84]	Invasive ductal or lobular carcinoma	Egypt	80	30	30 of 80	Yes	30 of 80	No		No			BamH1‐W region
Golrokh Mofrad [85]	Invasive ductal, invasive lobular	Iran	59	11	4 of 59	Yes	4 of 59	No		No			EBNA1
Mohammad‐izadeh [86]	Ductal, other	Iran	80	80	6 of 80	No		No		Yes	6 of 80	LMP1	LMP1
Morales‐Sanchez [87]	Infiltrating ductal, in situ ductal, others	Mexico	86	65	4 of 86	Yes	4 of 86	No		No			BamHI‐W region
Naushad [88]	Invasive ductal, in situ, other	Pakistan	250	15	61 of 250	Yes	61 of 250	No		No			EBNA2
Pai [89]	Invasive ductal, metaplastic	India	83	7	25 of 83	No		Yes	25 of 83	No			EBER
Reza [90]	Invasive ductal carcinoma	Iran	100	100	18 of 100	Yes	18 of 100	No		No			EBV DNA, EBER
Richardson [91]	Infiltrating ductal carcinoma	New Zealand	70	70^a^	24 of 70	Yes	24 of 70	No		No			EBNA1
Sharifpour [92]	Ductal	Iran	37	35	10 of 37	Yes	10 of 37	No		No			EBNA3C
Torfi [93]	Unspecified	Iran	46	46	2 of 46	Yes	2 of 46	No		No			EBNA1
Yahia [94]	Invasive ductal, invasive lobular, carcinoma in situ	Sudan	92	50^a^	49 of 92	Yes	49 of 92	Yes	18 of 18	No			EBNA4, LMP1, EBER
Zekri [95]	Primary invasive	Egypt and Iraq	90	40	39 of 90	Yes	39 of 90	Yes	32 of 39	Yes	23 of 34	LMP1	EBER, EBNA1, LMP1
Cervical													
Al‐Thawadi [96]	Not reported	Syria	44	0	15 of 44	Yes	15 of 44	No		Yes	Not reported	LMP1	LMP1, EBNA1
Chavoshpour‐Mamaghani [97]	Squamous cell and adenocarcinoma	Iran	61	179	7 of 61	Yes	7 of 61	No		No			EBNA1, LMP1
Leukaemia and other haematologic													
Guan [98]	Acute leukaemia (ALL & AML)	China	185	37	64 of 185	Yes	64 of 184	No		No			BamHI‐W region
Marin [99]	Adult T‐cell leukaemia/lymphoma and NK‐cell lymphomas	Argentina	22	0	9 of 22	No		Yes	9 of 22	No			EBER
Morales‐Sanchez [100]	Paediatric ALL	Mexico	70	0	10 of 70^b^	Yes	10 of 70	No		No			BamHI‐W region
Sehgal [101]	ALL	India	25	30	8 of 25	Yes	8 of 25	No		No			BamHI‐W region
Tarrand [102]	CLL	USA	135	98	19 of 135	Yes^c^	19 of 135	No		No			LMP1
Visco [103]	CLL	Italy	220	41	129 of 220	Yes	129 of 220	No		No			EBNA1
NHL													
Abdelrahim [104]	NHL of the oral/maxillofacial region	Malaysia	14	0	0 of 14	No		Yes	0 of 14	No			EBER
Kim [105]	Mixed NHL	South Korea	80	0	20 of 80	No		Yes	20 of 80	Yes	2 of 9	LMP1, EBNA2	EBER, LMP1, EBNA2
d'Amore [106]	Mixed NHL	Denmark	520	0	66 of 520	No		Yes	66 of 520	Yes	17 of 65	LMP1	EBER1/2, BHLF, LMP1
Jaseela [107]	Mixed NHL	India	67	0	7 of 67	No		No		Yes	7 of 67	LMP1	LMP1
Noorali [108]	T‐cell NHL	Pakistan	92	0	51 of 92	Yes	51 of 92	Yes	33 of 51	No			gp220, EBER1
Ohshima [109]	B‐cell and T‐cell NHL (nodal)	Japan	106	13	52 of 106	Yes	24 of 106	Yes	52 of 106	Yes	10 of 106	LMP1, EBNA2, vIL‐10	EBV‐W, EBER1, LMP1, EBNA2, BALF5
Prostate													
Nahand [110]	Acinar adenocarcinoma, ductal adenocarcinoma, squamous cell	Iran	67	40	33 of 67	Yes	33 of 67	No		No			LMP1, LMP2, EBER1, EBER2, BALF5
Testicular													
Fend [111]	Classical seminoma, spermatocytic seminoma, mixed/non‐seminomatous	Austria	21	5	4 of 21	Yes	4 of 21	Yes	2 of 4^d^	Yes	0 of 10	LMP1	EBER1/2, LMP1
Shimakage [112]	Seminoma, embryonal carcinoma	Japan	27	25	27 of 27	Yes	12 of 14	Yes	3 of 19^e^	Yes	8 of 9	EBNA2, LMP1	BamHI‐W, EBER, EBNA2, LMP1

Abbreviations: ALL, acute lymphoblastic leukaemia; AML, acute myeloid leukaemia; BALF5, BamHI‐A fragment containing the fifth leftward open reading frame; BamHI, a restriction enzyme derived from Bacillus amyloliquefaciens H; BHLF, BamHI H fragment leftward open reading frame; BHRF, BamHI H fragment rightward open reading frame; CLL, chronic lymphocytic leukaemia; DCIS, ductal carcinoma in situ; EBER, EBV‐encoded small RNAs; EBNA, EBV nuclear antigen; EBV, Epstein‐Barr virus; EBV‐W, EBV W region DNA; gp, glycoprotein; IHC, immunohistochemistry; IL, interleukin; ISH, in situ hybridisation; LMP, latent membrane protein; ML, malignant lymphoma; mT/NK, mature peripheral T‐cell and NK‐cell lymphomas; NASBA, nucleic acid sequence‐based amplification; NHL, non‐Hodgkin lymphoma; NK, natural killer; PCR, polymerase chain reaction; vIL, viral interleukin.

^a^ Study used paired cancerous and normal tissues from same individuals.

^b^ EBV only detected by nested PCR. Authors conclude this does not support a role for EBV in leukemagenesis.

^c^ Isothermal NASBA was used instead of PCR.

^d^ EBERs only detected in reactive lymphocytes, tumour cells were negative.

^e^ In house BamHI‐W ISH probe stained 27 of 27 tumour samples.

## Appendix 7 Country‐by‐Country Age‐Standardised Incidence Rates of Cancers of Interest

Maps of the country‐by‐country incidence rates of the cancers of interest, where data were available from IHME. (a) ALL, (b) AML, (c) breast, (d) cervical, (e) CLL, (f) CML, (g) NHL, (h) other leukaemia, (i) prostate, (j) testicular. White indicates data not available. Breast cancer data are for males and females. Prostate, cervical and testicular estimates are single‐sex in both instances. ALL, acute lymphoblastic leukaemia; AML, acute myeloid leukaemia; ASIR, age standardised incidence rates per 100,000 person years; CLL, chronic lymphocytic leukaemia; CML, chronic myeloid leukaemia; IHME, Institute for Health Metrics and Evaluation; NHL, non‐Hodgkin lymphoma.
